# A glimpse of an ancient agricultural ecosystem based on remains of micromammals in the Byzantine Negev Desert

**DOI:** 10.1098/rsos.171528

**Published:** 2018-01-10

**Authors:** Tal Fried, Lior Weissbrod, Yotam Tepper, Guy Bar-Oz

**Affiliations:** Zinman Institute of Archaeology, University of Haifa, Mount Carmel 3498838, Israel

**Keywords:** palaeozoology, Byzantine period, Southern Levant, anthropogenic impacts, micromammals

## Abstract

It is widely believed that Byzantine agriculture in the Negev Desert (fourth to seventh century Common Era; CE), with widespread construction of terraces and dams, altered local landscapes. However, no direct evidence in archaeological sites yet exists to test this assumption. We uncovered large amounts of small mammalian remains (rodents and insectivores) within agricultural installations built near fields, providing a new line of evidence for reconstructing anthropogenic impact on local habitats. Abandonment layers furnished high abundances of remains, whereas much smaller numbers were retrieved from the period of human use of the structures. Digestion marks are present in low frequencies (20% of long bones and teeth), with a light degree of impact, which indicate the role of owls (e.g. *Tyto alba*) as the principal means of accumulation. The most common taxa—gerbils (*Gerbillus* spp.) and jirds (*Meriones* spp.)—occur in nearly equal frequencies, which do not correspond with any modern Negev communities, where gerbils predominate in sandy low-precipitation environments and jirds in loessial, higher-precipitation ones. Although low-level climate change cannot be ruled out, the results suggest that Byzantine agriculture allowed jirds to colonize sandy anthropogenic habitats with other gerbilids and commensal mice and rats.

## 1. Introduction

**Figure 1.**
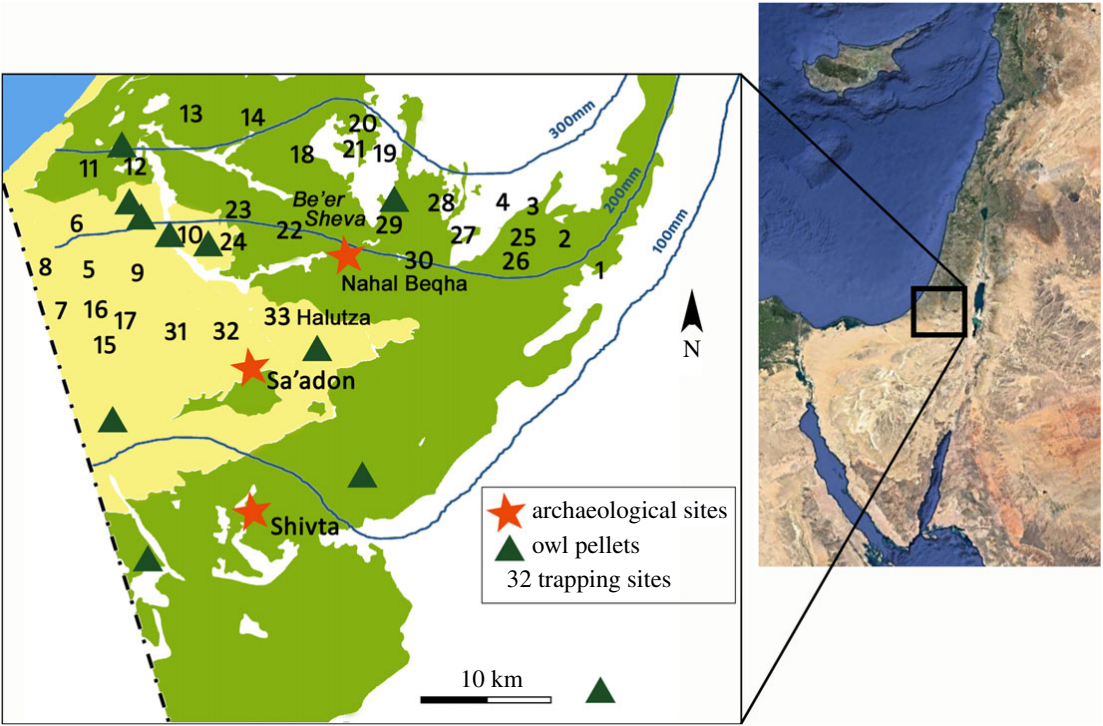
Map of the Negev showing the location of the study sites and sampling locations of modern data on micromammals in the region (trapping: [4], owl pellets: [5–9], archaeological: this study, [10,11]). Precipitation isohyets from the Meteorological Service of Israel (http://www.ims.gov.il/ims/all_tahazit/). Soil distribution data from Dan *et al*. [12]. Inset map data: Google, SIO, NOAA, US Navy, NGA, GEBCO and Image Landsat/Copernicus.

The Negev Desert features extensive remains of ancient human settlement and agricultural activities dating primarily to the Byzantine period (fourth to seventh century Common Era; CE). The settled expanse with an estimated 40 km^2^ of cultivated land area [1–3] extended south of the present day 200 mm isohyet, located in the Be'er Sheva Valley (figure 1). Archaeological remains reveal the presence of a major urban centre at the site of Halutza, six additional village-scale settlements and hundreds of farms [13]. There is also abundant evidence of widespread construction of agricultural terraces and dams for collecting flood waters and alluvium [1,14–16]. Written sources from the Byzantine period comprise descriptions of the local agricultural produce from fields and orchards of the Negev, including extensive grape cultivation and wine production [13,17,18]. Among the agricultural installations strewn across this landscape are dovecotes (columbaria), which were built near fields to produce fertilizers, enriching the nutrient-poor desert soils [19].

It has been suggested that Byzantine agricultural development in the Negev Desert altered local landscapes through farming, livestock grazing and settlement activities [20]. Byzantine activities in the Negev were unprecedented in scale and did not recur in the region until the modern era, representing a human achievement drawing on ingenuity and technological solutions in a challenging environment. Incentives for the Byzantine Negev growth spurt may have involved external funding and supply of human resources initiated by the Byzantine Empire [19], or reflected local initiatives based on long-term accumulation of knowledge on the Negev environment and experience in implementing runoff water agriculture [21]. It has also been argued that climatic change or stabilization initially aided human efforts in the Byzantine Negev, but that subsequent climatic deterioration led to cultural decline in post-Byzantine times [22–24]. However, palaeo-climate reconstructions have been based on off-site proxies such as Dead Sea and ground water level shifts or cave speleothem records [24,25]. Because of an absence of palaeo-environmental data obtained directly from archaeological contexts in the area, and which can be linked reliably to either climate change or anthropogenic influence, it remains difficult to test alternative scenarios for explaining the rise and fall of Byzantine agriculture in the Negev.

Remains of micromammals (rodents and insectivores) from archaeological sites have been used for reconstructing environmental conditions surrounding ancient settlements [26–28]. Small rodents and shrews in the Negev, which include a preponderance of dry-adapted species of gerbils and jirds (Gerbillidae), have generally adjusted to conditions of water and food shortages along with the high temperatures. The main water supply for most species in this arid environment is from ingested food [29]. With their dryland adaptations, most of these species remain hidden during the day in cooler and more humid shelters. Micromammals are present in a wide variety of habitats in the Negev and are considered especially sensitive to environmental variation. Their community structure is thought to respond to factors such as variation in precipitation or soil type and their influence on vegetation covers over relatively small scales [30]. Research based on live-trapping of small mammals in the Negev and analysis of prey remains in pellets of owls (Strigiformes) demonstrated consistent habitat associations of local species. Both habitat features (e.g. soil type, rockiness and proportion of vegetation cover) and proximity to different types of anthropogenic habitats were shown to influence species composition [30,31]. Data from owl pellets also showed a strong impact of recent anthropogenic landscape change on community composition owing to modern settlement and agriculture [5–8,10]. Research on long-eared owl (*Asio otus*) pellets conducted near modern agricultural settlement in the Negev suggests that anthropogenic environments are characterized by high frequencies of commensal species of mice and rats (*Mus musculus domesticus* and *Rattus rattus*), reaching more than 50% [9]. Leader *et al*. [9] also found that long-eared owl pellets in areas of sandy soils contained an abundance of gerbils (*Gerbillus* spp.), whereas those in loess soils contained more species of jirds (*Meriones* spp.). Other studies of owl pellets showed that in more remote parts of the Negev, prey composition is more diverse and consists mainly of wild species (non-commensal), mainly gerbilids [6–8,10].

Architectural remains of ancient settlements and agricultural systems on their own provide only indirect evidence for past environmental conditions in the Negev. Micromammalian remains collected directly from archaeological contexts can provide a useful proxy for reconstructing environmental conditions during the Byzantine period. Through the application of known aspects of the ecology and habitat requirements of identified taxa, we employ this type of proxy record in order to examine the impact of Byzantine agricultural activities in the Negev. We investigate taxonomic composition of the archaeological assemblages and conduct multivariate quantitative comparisons between our archaeological data on micromammals and modern data on micromammalian communities collected from both owl pellets and trapping studies in different types of habitats across much of the Negev (figure 1). We consider environmental aspects of substrate (soil type and rocky) and precipitation levels, both affecting vegetation cover.

### 1.1. Site and settings

Micromammalian remains were uncovered in three dovecotes from the Byzantine sites of Shivta and Sa'adon, located in the heart of the ancient Negev settlement (figure 1). The site of Shivta is located on a low ridge bonded by two wadies (Zeitan and Qorhah). Adjacent to this site, a round-shaped dovecote (structure 6) was excavated in 2000–2004, yielding a thick layer of debris which was associated with the stage of human use of the structure [32]. This was indicated by large amounts of pigeon (*Columba livia*) bones and dung [33,34]. Two additional dovecotes were excavated in 2011 near the site of Sa'adon, located approximately 10 km north of Shivta in a dune area [35]. A human occupation layer similar to the one uncovered in structure 6 of Shivta was exposed in a square-shaped dovecote in Sa'adon (structure B). In a second round-shaped dovecote discovered near Sa'adon (structure A), especially large amounts of micromammalian remains were retrieved from two loci (107 and 108), which appear to have accumulated after human abandonment of the structure. The absence of a clear occupation layer with pigeon bones and dung indicates planned human abandonment of the structure. The presence of wall and ceiling debris in the enclosed fill of the structure suggests its gradual collapse over time in conjunction with the formation of the fill deposits.

## 2. Material and methods

**Figure 2.**
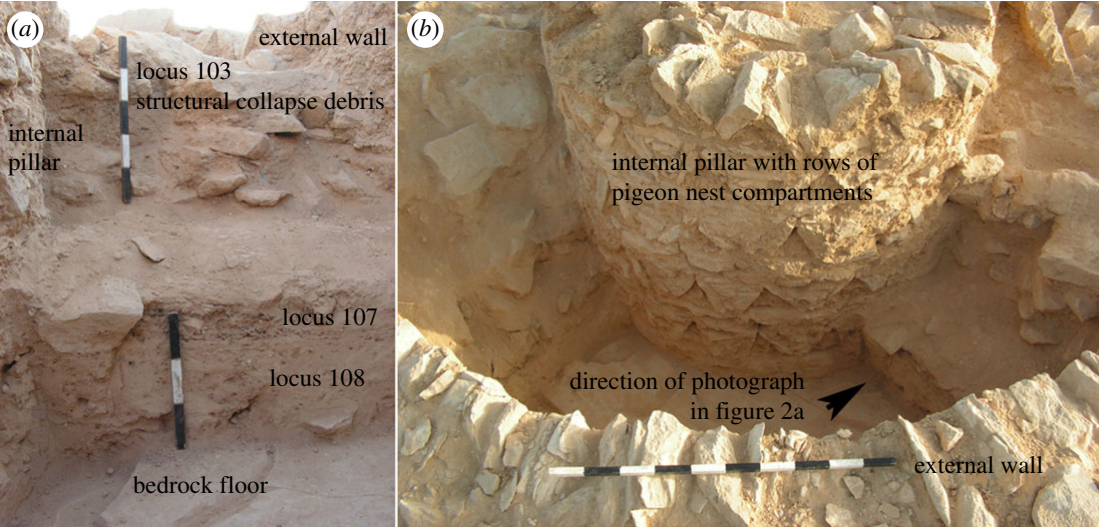
Field photographs of dovecote structure A of Sa'adon: (*a*) view of a section through the internal part of the structure, showing the 30 cm thick deposit with abundant micromammalian remains (loci 108 and 107), sandwiched between the bedrock floor and collapse debris from the upper part of the structure and (*b*) outside view of structure A, showing the location of the internal section (photographs by Yotam Tepper).

Excavations in the Shivta and Sa'adon dovecotes comprised exposure of trenches bisecting the structures. In structure A of Sa'adon, Byzantine period coins were uncovered near wall foundations indicating the time of construction [35]. A short succession, approximately 30 cm thick and composed of two layers (figure 2: loci 107 and 108), was exposed directly above the bedrock floor of this structure and below collapse debris from its upper part, including wall stones and roof slabs (figure 2: locus 103). This relatively thin stratigraphic sequence, lacking the pigeon bone and dung layer that was uncovered in the other two structures and containing large quantities of remains of micromammals, suggests accumulation which post-dates the time of human abandonment of structure A, but that in all likelihood occurred soon after this event. It is presumed that the site as a whole was permanently abandoned at the end of the Byzantine period (early seventh century CE), based on the extreme rarity of any findings from later periods [19]. In the structure 6 dovecote from Shivta, two rooms out of three were completely exposed and were excavated down to bedrock. Shards of sixth century CE cooking pots and lids found on the living surface suggest that this structure was also built in the Byzantine period [32]. Structure B of Sa'adon did not yield further dateable material.

Remains of micromammals were collected from fine-sieved (1 mm mesh) bulk soil samples from the excavations. The remains were laboratory sorted by skeletal elements. Taxonomic identification of molar teeth, maxillae and mandibles was conducted using the comparative collections of the Laboratory of Archaeozoology, University of Haifa and the National Natural History Collections of the Hebrew University in Jerusalem. Identified molars (and rare instances of maxilla and mandible counts) were employed as a proxy for taxonomic abundance, including a number of identified specimens (NISPs) and minimum number of individuals (MNIs) [36]. In addition, the Negev contains as many as two genera with six different species of Gerbillidae. An attempt was made to identify remains to the species level using molar tooth row length in comparison with published statistics on recent individuals [29,37], though sample sizes for complete tooth rows are small (*Gerbillus*: nine mandibles and one maxillae; *Meriones*: four mandibles and two maxillae), and do not allow clear-cut identification and quantification at the species level.

Skeletal element preservation was assessed to shed light on depositional history (taphonomy) of the assemblages. The minimum numbers of elements (MNEs) were reconstructed using Andrews' equation [38] for element relative abundance, expressed as a proportion of expected numbers of each element given the MNI in each assemblage. Teeth, jaws and long bones (NISP = 3538) were inspected microscopically (magnification of ×8–12.5) to identify modifications, such as digestion marks, which are owing to predator accumulation. Identified marks were recorded according to the level of their severity following Andrews [38]: 1—light, 2—moderate, 3—medium and 4–5—heavy/extreme. Age at death of individuals was determined based on tooth wear following Lidicker [39] for common mice and Osborn & Helmy [40] for Gerbillidae. Age was also assessed based on epiphyseal fusion in long bones, noting the presence of unfused epiphyses (sub-adult) and of fused ones (adult).

Finally, using multivariate exploratory ordination methods, we compared taxonomic composition in the dovecote assemblages to the data obtained from studies of modern Negev communities through live-trapping and analysis of owl prey conducted in different parts of the region. Exploratory statistical techniques of principal component analysis and correspondence analysis, suitable for use with frequency and abundance data types, respectively, allow us to compare multiple assemblages composed of several taxa each. In these analyses, we employ MNI calculations to represent taxonomic composition in the archaeological assemblages. These reconstructed figures should more closely correspond to the data format used in studies of both trapping and recent owl pellets, which are based on counts of individuals. Although the use of NISPs rather than MNIs is generally recommended in archaeozoological studies owing, in part, to issues of aggregation [36,41], in this study counting of MNIs in each assemblage was based on pooling material from a whole undifferentiated archaeological locus. Moreover, it has been suggested that consistent use of a restricted range of skeletal elements (e.g. only cranial elements) for taxonomic identification, as is often the case with studies of micromammalian remains, should greatly improve correspondence between MNI and NISP calculations [41].

Because we compare ancient and modern assemblages, each relying on different forms of quantification of species abundances (i.e. MNIs versus trapping densities), we also introduce a Gerbillidae index (*Meriones*/*Gerbillus*) as a way of standardizing data across assemblages for the purpose of direct quantitative comparisons. The statistical software past (PAleontological STatistics, v. 3.11) [42] was used for all statistical analysis and to compute species diversity using the Shannon H index. Univariate nonparametric statistics (*χ*^2^ test and Mann–Whitney), which do not subsume an assumption of normality, were also used in comparisons between archaeological assemblages (skeletal element distributions) and between different trapping habitats in terms of rodent densities. Standard descriptive statistics, including ranges, means and coefficients of variation (CV), were also provided where relevant.

## 3. Results

### 3.1. Taxonomic identification

Identification of cranial and dental remains (principally molars: NISP = 1051) indicates that skeletal elements from gerbils and jirds are most abundant in the assemblages, with relatively low numbers of specimens from: lesser Egyptian jerboa (*Jaculus jaculus*), house mouse (*M. musculus domesticus*), Asian garden dormouse (*Eliomys melanurus*), black rat (*R. rattus*), mole rat (*Spalax ehrenbergi*) and sand rat (*Psammomys obesus*), and the insectivore shrews (*Crocidura* sp. and *Suncus etruscus*) (table 1).

**Table 1.** Taxonomic composition in the different dovecote structures based on molar teeth (except where maxillae or mandibles outnumber molars).

	Sa'adon						Shivta			
	structure A—round (human abandonment)									
	locus 107		locus 108		structure B—square (human use)		structure 6 (human use)		Total	
taxa	NISP	MNI	NISP	MNI	NISP	MNI	NISP	MNI	NISP	MNI
Rodentia										
* Gerbillus* spp.	248	49	74	17	1	2^a^	1	1	324	67
* Meriones* spp.	364	50	134	17	3	1	1	1	502	69
* Jaculus* sp.	43	17	24	6	—	—	2	1	69	24
* Mus* sp.	7	5	4	2	7	3	1	1	19	11
* Eliomys melanurus*	3	4^b^	—	—	—	—	—	—	3	—
* Rattus rattus*	—	—	—	—	2	2	—	—	2	2
* Spalax ehrenbergi*	2	2	—	—	—	—	—	—	2	2
* Psammomys obesus*	—	—	2	1	—	—	—	—	2	1
Insectivora										
* Crocidura* sp.	111	21^b^	17	4^a^	—	—	—	—	128	—
* Suncus etruscus*	—	1^b^	—	—	—	—	—	—	—	—
total									1051	176
Shannon_H diversity	1.558		1.424		1.321		1.386			

^a^Maxilla counts.

^b^Mandible counts.

Taxonomic composition differs between assemblages collected from contexts of human occupation (structures B, 6) and abandonment (structure A, loci 107 and 108). The former contained a combined NISP of only 18 compared with a combined NISP of 1003 for the two abandonment layers of Sa'adon's structure A and was also considerably less rich in species (*N*_taxa_ = 4 compared with *N*_taxa_ = 6–8). In addition, commensal mice and rats seem to be associated more with the occupation layers than with the abandonment ones. Though common mice are present in all of the analysed contexts, their proportion is quite low (less than 2%) in the two abandonment layers and high (greater than 20%) in the occupation layers. One of the occupation contexts (structure B) also contained the only finds of a black rat. While sample sizes are low from layers associated with human use, the high relative abundance of the remains of commensal taxa suggests an important difference in mode of accumulation.

### 3.2. Taphonomy

**Figure 3.**
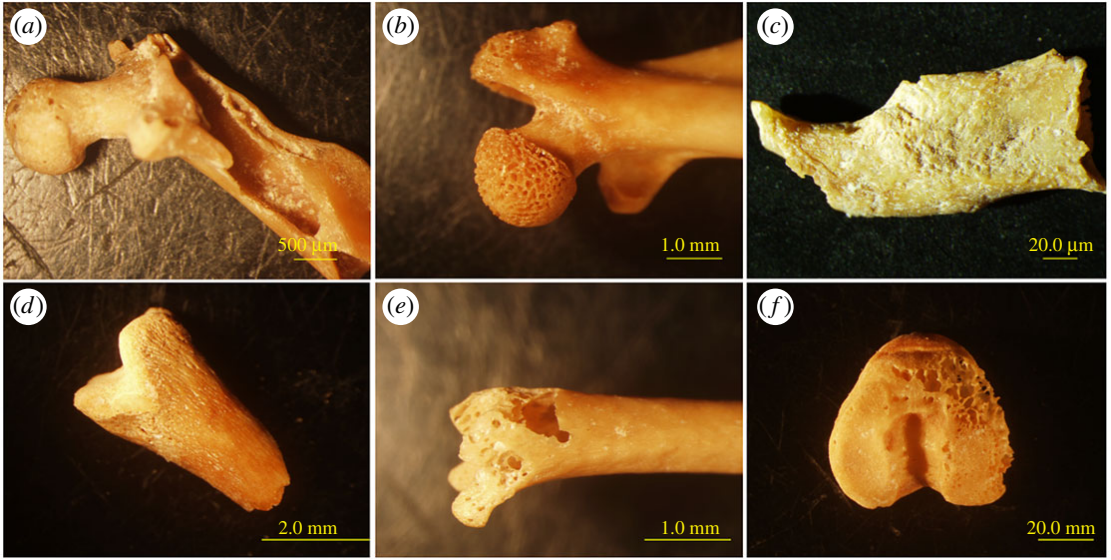
Typical digestion marks on skeletal remains of micromammals from the structure A dovecote in Sa'adon on elements of the femur (*a*,*b*,*d*,*e*), mandible (*c*) and tibia (*f*).

The bones are well preserved and most of them are complete and highly identifiable (e.g. percent of complete long bones = 59). All skeletal parts are present, including a few *clavicle* and *hyoid* bones, which are rarely found in archaeological sites [38]. A proportion of 61% of specimens that were examined (long bones, jaws and teeth; total NISP = 3538) show some form of modification. The presence of splitting, cracking, rounding, pitting and porosity on bones and teeth is indicative of the impact of digestion caused by a predator. Porosity of bony surfaces and structures (figure 3) is considered a unique indicator of digestion [38] and was discovered on 9% of the incisors teeth, 12% of the molar teeth and 31% of the long bones. The severity of most of the digestion marks was defined as light (42% of the incisor teeth, 39% of the molar teeth and 58% of the long bones). The frequency of digested bones (a combined average of 20% for long bones and teeth) suggests light–moderate severity, which is consistent with modifications observed from owl pellets ([38], table 3.16). In summary, both the frequency and severity of digestion are consistent with modifications observed in the skeletonized prey of owls.

**Figure 4.**
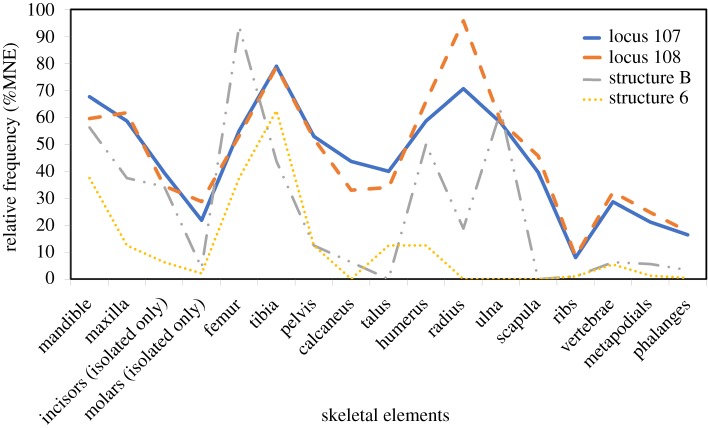
Skeletal element distribution patterns for the four dovecote assemblages, based on data in table 2.

In addition, possible signs of weathering (splitting, cracking, peeling, flaking and pitting), acidic soil corrosion (oxidation stains, punctures, peeling, flaking and pitting), root corrosion (grooves and pitting) and gnawing (grooves and punctures) were noted in relatively low frequencies. The relative frequency of the different skeletal elements was calculated according to Andrews' equation [38, p. 45], showing a good state of preservation, especially of the jaws and long bones, in all four assemblages (approx. 38–68%). Smaller elements such as ribs and phalanges survived less well, ranging in relative frequencies between 0% and 18% (table 2 and figure 4). In the relatively small assemblages from structures B and 6, some of the skeletal elements are altogether missing (e.g. radius, ulna and scapula), probably reflecting a sample size effect.

**Table 2.** NISPs and %MNEs by dovecote structures.

	locus 107		locus 108		structure B		structure 6	
element	NISP	%MNE	NISP	%MNE	NISP	%MNE	NISP	%MNE
mandible	203	67.67	56	59.57	9	56.25	3	37.50
maxilla	176	58.67	58	61.70	6	37.50	1	12.50
incisors (isolated only)	237	39.50	65	34.57	11	34.38	1	6.25
molars (isolated only)	394	21.89	162	28.72	4	4.17	1	2.08
femur	165	55.00	50	53.19	15	93.75	3	37.50
tibia	237	79.00	74	78.72	7	43.75	5	62.50
pelvis	159	53.00	49	52.13	2	12.50	1	12.50
calcaneus	131	43.67	31	32.98	1	6.25	0	0.00
talus	120	40.00	32	34.04	0	0.00	1	12.50
humerus	176	58.67	62	65.96	8	50.00	1	12.50
radius	212	70.67	90	95.74	3	18.75	0	0.00
ulna	174	58.00	55	58.51	10	62.50	0	0.00
scapula	119	39.67	43	45.74	0	0.00	0	0.00
ribs	289	8.03	95	8.42	2	1.04	1	1.04
vertebrae	1,375	28.65	485	32.25	16	6.25	7	5.47
metapodials	636	21.20	233	24.79	9	5.63	1	1.25
phalanges	1481	16.46	499	17.70	16	3.33	1	0.42
total (average %MNE)	6284	44.69	2139	46.16	119	25.65	27	12.00

A *χ*^2^ test of association for NISP-based skeletal frequencies was conducted between the two loci of structure A in Sa'adon (loci 107 and 108). This analysis indicates that the difference in skeletal frequencies between the two assemblages is not significant (*χ*^2^_16_ = 1923.9; *p* < 0.01). Element frequency distributions based on MNE calculations also demonstrate similarity with other data on skeletal element frequencies of nocturnal raptors which were studied by Andrews [38], where relative frequencies likewise tend to be high for the jaws and long bones (approx. 40–90%) and low for small skeletal parts such as ribs and phalanges (approx. 6–35%) ([38], figs 3.2, 3.3 and append. table 12). The analysis of additional skeletal element preservation indices, including the proportion of crania to post-cranial elements and of distal to proximal limb bones (106% and 104%, respectively), shows correspondence to ranges obtained from studies of modern nocturnal raptor prey assemblages (74–164% and 52–105%, respectively) ([38], table 3.2).

### 3.3. Age assessment

We documented intermediate levels of wear for the majority of molar teeth (72%), indicating that most of the individuals are sub-adults or adult, but not old. Teeth of old individuals (NISP = 6) belong to commensal house mice. The analysis of epiphyseal fusion showed that most of the long bones (57%) are fused in only one of the epiphysis. This supports the observation of an intermediate level of tooth wear and the sub-adult or adult age of most individuals in the assemblage.

### 3.4. Comparison to modern assemblages

#### 3.4.1. Trapping assemblages

**Figure 5.**
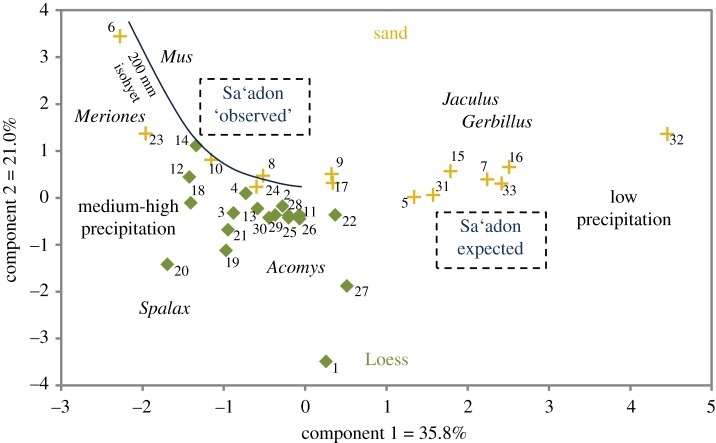
Principal component analysis of taxonomic composition in modern live-trapping studies across the Negev (see site locations in figure 1) [4]. Component 1 plots sites in areas of low annual precipitation (less than 200 mm) on the right and those in areas of medium–high annual precipitation (greater than 200 mm) on the left. Along component 2, sites in sandy soils (sandy dunes and regosols and arid brown soils; yellow plus signs) plot on top, whereas those in loess soils (loessial arid brown soils and serozems and brown lithosols; green rhombuses) mostly on the bottom of the graph. Soil types from Dan *et al.* [12].

The assemblages from the square dovecote of Sa'adon (structure B) and the dovecote of Shivta (structure 6) have small sample sizes and the use of quantitative comparisons is limited. Principal component analysis of taxonomic composition of micromammals in modern trapping studies across 3300 km^2^ in the Northern Negev [4] (figures 1 and 5) shows that these assemblages group both by precipitation levels (along component 1) and soil type (along component 2). Component 1 also aligns with a trend of increasing gerbil (*Gerbillus* spp.) but decreasing jird (*Meriones* spp.) densities from left to right, with decreasing precipitation. Under modern conditions, the expected position of the archaeological samples from structure A of Sa'adon would be with assemblages on the right (figure 1: 5, 7, 15–16 and 31–33), which are characterized by relatively high gerbil densities (2.8–8.4 individuals ha^−1^), low precipitation (less than 200 mm annually) and sandy habitat (table 3). The Gerbillidae index (jirds/gerbils) in five of these seven trapping assemblages, where jirds are scarce but not altogether absent, has a low mean value of 0.07, whereas the same index in the Sa'adon assemblages is considerably higher (locus 108 = 1; locus 107 = 1.02) owing to nearly equal frequencies of the two taxa (table 3). The Sa'adon assemblages with their higher-than-expected jird frequencies should fall in the top left part of the graph (figure 5; archaeological data could not be plotted in the same ordination space with trapping data owing to the fundamental difference in the nature of these datasets), where collection sites are located in a more northerly position than those of Sa'adon, abutting the 200 mm precipitation isohyet (figure 1: 6, 8, 10 and 23–24). Taking the trapping data on varying gerbilid densities, jird densities are significantly higher on the left side of the graph within a higher-precipitation area (2.2–5.6 individuals ha^−1^) than on the right (0.3–0.9 individuals ha^−1^; Mann–Whitney *U*-test *z* = −2.538, *p* = 0.01), and gerbil densities are lower, though not significantly so (0.9–1 individuals ha^−1^; *z* = −1.911, *p* = 0.06). It is the data from collection sites near the 200 mm isohyet (table 3, italic values in cells) that are consistent with the ‘observed’ position of the Sa'adon assemblages, rather than the data from the low-precipitation sites which characterize the Sa'adon area today (figure 5).

**Table 3.** Basic statistics and the Gerbillidae index describing data from trapping studies, analyses of recent owl pellet assemblages and Byzantine owl assemblages of the Negev. (Cases with zero values are excluded from calculations. In the fourth column presenting data on high precipitation, loess soil material 15 of the 19 trapping sites contain zero *Gerbillus* and the Gerbillidae index could not be accurately determined.)

		precipitation/soil			
		low/sandy	low/loessial	medium–high/loessial	high/loessial
trapping densities (individuals ha^−1^)					
* Gerbillus*	no. of sites	7	7	2	4
	minimum	2.8	1.32	0.9	0.3
	maximum	8.4	6.88	1	2.8
	mean	1.91	3.73	—	1.65
	CV	29%	51%	—	55%
* Meriones*	no. of sites	5	6	5	17
	minimum	0.3	0.33	2.2	0.3
	maximum	0.9	1.5	5.6	3
	mean	0.48	0.86	3.2	1.06
	CV	24%	46%	43%	90%
index Gerbillidae					
* *trapping	no. of sites	5	6	2	4
	minimum	0.04	0.14	*2.44*	0.05
	maximum	0.18	0.39	*2.5*	0.53
	mean	0.07	0.21	—	0.26
	CV	72%	41%	—	87%
archaeological assemblages: based on present day (observed) precipitation/soil conditions					
* *Sa'adon (structure A)	locus 108	1			
	locus 107	1.02			
* *Nahal Beqha				3.94	
modern owl pellet assemblages					
	no. of sites	6			2
	minimum	0.05			29.33
	maximum	0.43			34.56
	mean	0.25			—
	CV	63%			—

**Figure 6.**
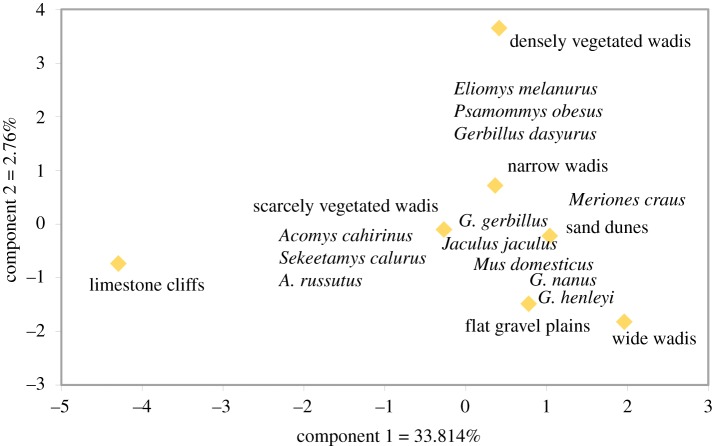
Principal component analysis of taxonomic composition in a modern live-trapping study in different habitat types in the Ramon Crater, Negev Highlands, based on density data ([30], table 2).

On a smaller spatial scale within the Negev, high gerbil densities (1.32–6.88 individuals ha^−1^) and low jird densities (0.33–1.50 individuals ha^−1^) are noted in a series of microhabitats in the Ramon Crater (*N* = 7; table 3), located in an arid area south of the 100 mm precipitation isohyet but in a loess soil region (see [30], table 2). As can be expected for arid areas, the jird to gerbil index in these assemblages is relatively low (0.14–0.39) in comparison with data from sites with medium–high precipitation (2.44–2.50). It is interesting that the Gerbillidae index in the Ramon Crater sites within a loess area is higher than in other trapping sites with low precipitation, which are located in sandy soils (table 3). A principal component analysis of the Ramon Crater trapping data shows that the common taxa in the Sa'adon assemblages (gerbils, jirds, jerboa and common mice) occur in a wide range of habitat types, including wadis, sand dunes and gravel plains, positioned on the right side of the ordination space in figure 6. This pattern points to the likelihood that a variety of local microhabitats contributed to the formation of the archaeological dovecote assemblages. These results are an indication that locally increased water budgets allowed communities of small mammals with relatively high abundances of both gerbils and jirds to extend into an arid part of the Negev in the Byzantine period.

#### 3.4.2. Owl pellet assemblages

**Figure 7.**
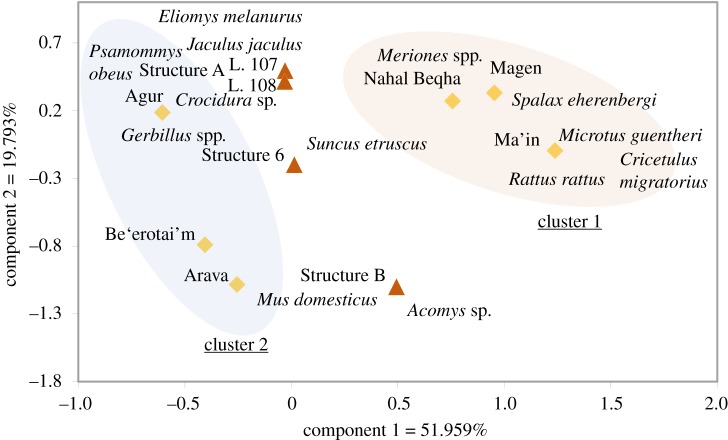
Correspondence analysis of taxonomic composition comparing our archaeological dovecote samples to modern owl pellet assemblages collected within different types of habitats and climate and soil zones in the Negev (modern: [23–26] and archaeological: [27,41]).

Using abundance data with a correspondence analysis technique, taxonomic composition from the four archaeological dovecote assemblages was compared with the composition of recent owl pellet assemblages collected within different parts of the Negev: Kibbutz Magen [6], Nahal Beqha [11], Wadi Zarqa Ma'in 1 [10], Holot Agur [8], Be'erotayim [5] and Arava Valley [7]. All assemblages were collected from active nests of barn owls, except for two: one from Nahal Beqha, which was collected from a Byzantine dovecote in the Negev where the accumulating agent was not identified [11], and the other from Wadi Zarqa Ma'in 1, which was collected in an open sinkhole in Jordan found to contain ancient prey remains [10]. Two distinct clusters can be detected along component 1 of the ordination space (figure 7): cluster 1 includes assemblages which have relatively high proportions of jirds (45–86%) and occur in areas of higher precipitation (greater than 200 mm annually) and mainly loess soil cover, whereas cluster 2 includes assemblages with greater frequencies of gerbils (53–77%) which occur in areas of lower precipitation (less than 200 mm annually) and mainly sandy soil. The dovecote assemblages fall squarely in between clusters 1 and 2 in figure 7, indicating again that their composition of nearly equal abundances of gerbils and jirds does not easily correspond with what can be expected in the Sa'adon locality based on modern data. This is also the case with the Gerbillidae index, which is high in the cluster 1 Magen site (34.56) but low in the three cluster 2 sites (0.04–0.4).

## 4. Discussion

### 4.1. Taphonomy and predation

The occurrence of digestion marks detected on the skeletal remains and the pattern of relative frequencies of skeletal elements indicate the role of predators in the accumulation of micromammals in the dovecotes. The frequency of occurrence and severity of these digestion marks accord with what can be expected from nocturnal raptors that tend to inflict only light degrees of damage to the remains of their prey [38]. These digestion marks together with the large quantities of remains in the assemblages from the round dovecote, structure A in Sa'adon, and high taxonomic diversity are all highly characteristic of assemblages accumulated by nocturnal raptors. However, skeletal remains from the use stages in structures B and 6 also show the presence of digestion marks, including on bones that belong to the commensal species mice and rats. It is possible that raptors entered these two structures soon after human abandonment and deposited the remains of their prey, which either became mixed with the remains of commensals from the use stage or account for the totality of the assemblages found in the structures. In this scenario, the seemingly marked presence of commensal species in the use stage could be the result of collection by the owls from an anthropogenic environment which was within their hunting range. Both barn owls and long-eared owls have been recorded within modern day human settlements in the Negev [9,43], and are widespread nocturnal raptors, which tend to inflict only light levels of digestion on the remains of their prey [38]. These two species of owl tend to focus on small mammals, mainly rodents and shrews, as their main source of prey in different parts of the world [44,45], though long-eared owls in the Negev show a greater tendency to switch to avian prey in situations of competition for food or seasonal prey shortages [43]. The differences in prey composition between barn owls and long-eared owls in the Negev were not significant, suggesting that prey selection for these groups of individuals was mainly related to natural abundances of prey within the hunting ranges [43].

Both tooth wear and epiphyseal fusion data indicate that most of the individuals in the dovecote assemblages are sub-adult or adult, but not very young or old. Such individuals are old enough to disperse from the parental nest, but may be inexperienced in terms of predator avoidance and are thus expected to be found in prey assemblages in high numbers [46].

Worn teeth of old individuals were found in both abandonment and use stages in the dovecotes of Sa'adon and belonged to house mice. Weissbrod *et al*. [47] suggested that old individuals will be found in commensal rodent communities and much less common in free-living communities, because longevity is extended among commensal rodents with high access to food resources and reduced pressure from predators. It is possible that house mice inhabited the dovecotes as commensals, whereas most other species in the dovecote assemblages were introduced as the remains of owl prey. However, as noted above, digestion marks were also detected on bones of house mice, suggesting that they too were raptor prey. Thus, commensal mice may have been preyed upon by owls within anthropogenic environments during the time when dovecotes were in use as well as after their abandonment. It is not possible to say at this time whether the abandonment of these dovecotes coincided with general abandonment of the surrounding landscape and settlement, or whether some form of less intense human use of the landscape lacking exploitation of pigeon manure fertilizer continued. However, stratigraphic indicators together with the size of our assemblages (MNI = 176) suggest rapid accumulation of the assemblages (see [28] for discussion of a similar short-term accumulation scenario), which probably came soon after human abandonment of the structures.

### 4.2. Taxonomic composition and environmental reconstruction

Layers belonging to the stage of Byzantine use of the structures are characterized by few skeletal remains of micromammals and low taxonomic diversity. This probably reflects habitual maintenance and cleaning activities of the dovecotes during the time of use and sealing of the structure opening as a way to control pests [48]. The species of micromammals found in the stages of human use could have accumulated within their living environment, as commensal species. In general, house mice and rats remains were more abundant in the use deposits than in the post-use or abandonment ones, though sample sizes in the former layers are small. These common commensal species in the use layers occur together with remains of other species such as gerbils and jirds. Deposits belonging to the stages of abandonment of the structures (structure A, loci 108 and 107), in contrast, are characterized by much larger samples of skeletal remains and high taxonomic diversity. These more diverse assemblages probably reflect accumulation during a relatively short time span and soon after the time that structure A was abandoned and before its final collapse.

Multivariate comparisons of the dovecote assemblages with modern data on taxonomic composition and environmental variables from modern studies (trapping and analysis of modern owl pellets) suggest that the taxonomic composition of the archaeological assemblages is more similar to that found in habitats elsewhere in the Negev than in the vicinity of Sa'adon today. However, our data also point to some change in the structure of these communities and potentially in environmental conditions between the Byzantine period and those present in the area of Sa'adon. This shift could have been owing to a climatic fluctuation or a change in the intensity of anthropogenic landscape use.

High abundances of gerbilid species (*Gerbillus* spp. and *Meriones* spp.) found in the dovecotes also characterize the different types of habitats investigated in trapping studies in the general area of Sa'adon and Shivta. Species from the Gerbilidae Family are generally adapted to arid climates and tend to inhabit deserts and steppe environments such as the characteristics of the Negev. The relatively diverse composition of the dovecote assemblages on the whole also indicates, however, the presence of species from a range of habitat types, rather than merely the immediate hilltop vicinity of the dovecotes. This leads us to suggest the existence of a mosaic of habitat patches, including more natural and anthropogenic ones, surrounding the dovecotes and forming part of the Byzantine agricultural landscape.

We employed the Gerbillidae index (jirds/gerbils) for tracking interactions between community structure and environmental conditions and for conducting a more fine-tuned assessment of the nature and magnitude of environmental change in the Negev. In the modern trapping data, the Gerbillidae index was significantly lower in the low-precipitation sandy areas where gerbils were considerably more abundant than jirds. The same general pattern was observed in modern owl data. However, the archaeological dovecote assemblages, which are likewise currently located in a low-precipitation area (and in the case of Sa'adon also in a sandy area), demonstrate an unexpected pattern for their present day environment, where the Gerbillidae index is considerably higher than would be expected from modern data in this area.

Modern owl pellet assemblages provide an important point of reference for interpreting the dovecote remains of micromammals as our taphonomic analysis strongly points to the involvement of owls in accumulating the archaeological material. Comparison between the ancient and modern assemblages shows that the Byzantine ones are intermediate in their community composition to those from areas with sand and loess substrates and low and high precipitation isohyets in the Negev. However, modern owl diets in the Negev, especially when collected near areas of rural agricultural or urban settlement, are notably biased towards high frequencies of commensal house mice and rats, probably collected from cultivated fields [9]. The scale of agricultural and settlement activities in modern times in the Negev is undoubtedly much greater than anything we might expect during the Byzantine period. Hence, we should expect a different type and magnitude of anthropogenic impact in the dovecote assemblages than those which characterize modern day, large-scale anthropogenic landscape influences. In the framework of our comparative study, this modern bias was, to some extent, circumvented through a focus on the use of the Gerbillidae index.

Anthropogenic agricultural activities of slope terracing, wadi damming and possibly field fertilizing, and also naturally increasing precipitation levels, could have elevated jird frequencies within a habitat that was less suitable for them. However, such environmental shifts may not have been sufficiently extensive to substantially elevate the abundances of common commensals and agricultural pests as house mice and rats, as often seen near modern farming settlements in the Negev [9]. Other studies based on trapping show that gerbils can occur in and around humanly settled areas, where they occur together with commensal house mice in urbanized areas ([49], table 16.1) and without the latter near isolated farms [31]. Gerbils were widely documented near urban buildings, and in gardens, rubbish dumps and groves. Near isolated farms gerbils were the most abundant species trapped and occurred together with spiny mice (*Acomys cahirinus*)—a known facultative commensal species—and lesser Egyptian Jerboa (*J. jaculus*) [31]. Thus, in the Byzantine period, the gerbil may have joined the typical commensal species house mouse and rat as well jirds to form a different commensal community than known today within modern large-scale agro-ecosystems.

## 5. Conclusion

The taxonomic composition of the dovecote micromammalian assemblages generally corresponds to what is known about community composition in different habitat types of the Negev today. It markedly differs, however, from what can be expected near modern agricultural settlements where especially high abundances of common commensal mice and rats are found. It stands to reason that the level of impact of Byzantine settlement and agricultural activities in the Negev was much reduced relative to that of modern and much more intensive agriculture in the region. Comparison of the archaeological assemblages with modern data based on trapping and owl pellet studies across different parts of the Negev, however, reveals higher-than-expected frequencies of jirds occurring together with abundant gerbils in the abandonment layers of Sa'adon. This distinguishes the ancient assemblages of Sa'adon from any known modern ones. High jird abundances characterize areas of the Negev with loess soils and higher precipitation, whereas in sandy drier areas gerbils abound. The high frequencies of both jirds and gerbils in the Sa'adon dovecote layers suggest the impact of Byzantine agriculture on the local landscape, which allowed jirds to inhabit this largely sandy and anthropogenic habitat, and to coexist in relatively high numbers with indigenous gerbils.

Based on our findings, species of jirds and gerbils formed a substantial component of ancient ecological communities of agro-ecosystems in the Negev, testifying to the environmental impact of Byzantine agriculture. Although Byzantine occupation of the Negev did not achieve the scale of modern human influence of industrialized farming, it undoubtedly exerted lasting impacts which altered ecological conditions and the structure of local plant and animal communities. Reconstructing the patterns of ancient human landscape transformation in greater detail will require additional trapping and owl pellet studies in modern non-industrial agricultural settings in the Negev. It is possible that localized anthropogenic impacts in the Negev were further augmented by regional climate fluctuations, though additional and more detailed palaeo-environmental data obtained directly from archaeological sites will be needed to assess this possibility.

## Data accessibility

All data and research materials supporting the results are in the article or have been made available online through Dryad.org (http://doi.org/10.5061/dryad.3h207) [11].
